# Differential Phosphorylation of Perilipin 1A at the Initiation of Lipolysis Revealed by Novel Monoclonal Antibodies and High Content Analysis

**DOI:** 10.1371/journal.pone.0055511

**Published:** 2013-02-06

**Authors:** Patrick M. McDonough, Dominique Maciejewski-Lenoir, Sean M. Hartig, Rita A. Hanna, Ross Whittaker, Andrew Heisel, James B. Nicoll, Benjamin M. Buehrer, Kurt Christensen, Maureen G. Mancini, Michael A. Mancini, Dean P. Edwards, Jeffrey H. Price

**Affiliations:** 1 Vala Sciences Inc, San Diego, California, United States of America; 2 Department of Molecular and Cellular Biology, Baylor College of Medicine, Houston, Texas, United States of America; 3 Zen-Bio, Inc, Research Triangle Park, North Carolina, United States of America; 4 Signal Transduction Program, The Sanford-Burnham Institute for Medical Research, La Jolla, California, United States of America; The University of Queensland, Australia

## Abstract

Lipolysis in adipocytes is regulated by phosphorylation of lipid droplet-associated proteins, including perilipin 1A and hormone-sensitive lipase (HSL). Perilipin 1A is potentially phosphorylated by cAMP(adenosine 3′,5′-cyclic monophosphate)-dependent protein kinase (PKA) on several sites, including conserved C-terminal residues, serine 497 (PKA-site 5) and serine 522 (PKA-site 6). To characterize perilipin 1A phosphorylation, novel monoclonal antibodies were developed, which selectively recognize perilipin 1A phosphorylation at PKA-site 5 and PKA-site 6. Utilizing these novel antibodies, as well as antibodies selectively recognizing HSL phosphorylation at serine 563 or serine 660, we used high content analysis to examine the phosphorylation of perilipin 1A and HSL in adipocytes exposed to lipolytic agents. We found that perilipin PKA-site 5 and HSL-serine 660 were phosphorylated to a similar extent in response to forskolin (FSK) and L-γ-melanocyte stimulating hormone (L-γ-MSH). In contrast, perilipin PKA-site 6 and HSL-serine 563 were phosphorylated more slowly and L-γ-MSH was a stronger agonist for these sites compared to FSK. When a panel of lipolytic agents was tested, including multiple concentrations of isoproterenol, FSK, and L-γ-MSH, the pattern of results was virtually identical for perilipin PKA-site 5 and HSL-serine 660, whereas a distinct pattern was observed for perilipin PKA-site 6 and HSL-serine 563. Notably, perilipin PKA-site 5 and HSL-serine 660 feature two arginine residues upstream from the phospho-acceptor site, which confers high affinity for PKA, whereas perilipin PKA-site 6 and HSL-serine 563 feature only a single arginine. Thus, we suggest perilipin 1A and HSL are differentially phosphorylated in a similar manner at the initiation of lipolysis and arginine residues near the target serines may influence this process.

## Introduction

**Figure 1:**
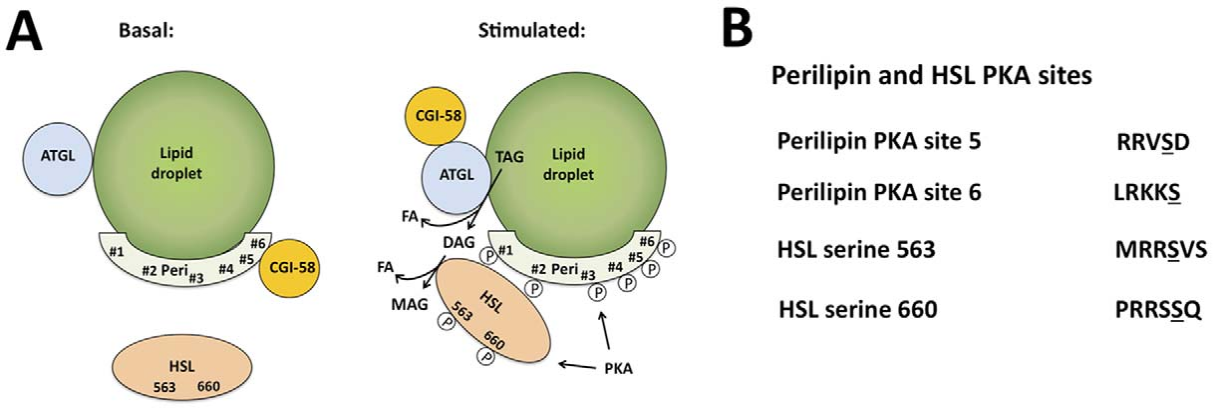
Regulation of lipolysis in adipocytes. A, A current model for the hormonal regulation of lipolysis initiation is shown. Proteins depicted include perilipin 1A (Peri), Hormone Sensitive Lipase (HSL), Adipocyte Triglyceride Lipase (ATGL), CGI-58, and PKA. Lipid species depicted include triacylglycerol (TAG), diacylglycerol (DAG), monoacylglycerol (MAG), and fatty acid (FA). Under basal conditions, perilipin and HSL are unphosphorylated and HSL is found throughout the cytoplasm. Stimulation of lipolysis involves activation of PKA, phosphorylation of perilipin 1A and HSL, release of CGI-58 from perilipin, binding of CGI-58 to ATGL, and translocation of HSL to perilipin. TAG is sequentially processed to DAG by ATGL and to MAG by HSL with FA released at each step. B, Amino acid sequences are shown for perilipin 1A PKA site 5, and PKA site 6, and for HSL serine 563 and serine 660. The target serine in each sequence is underlined.

Lipid droplets are cellular organelles consisting of a neutral lipid core of triacylglycerides (TAGs), surrounded by a phospholipid membrane and a suite of proteins, which regulate lipid metabolism [1]. Lipolysis is a key metabolic process whereby TAGs in the lipid droplets are processed by lipases to release fatty acids for ß-oxidation. A current model for the initiation of lipolysis [2] in adipocytes is presented in Figure 1. The protein perilipin 1A (PLIN1) was the founding member of the five gene perilipin family [3]. Perilipin 1A is tightly associated with the cytoplasmic side of the lipid droplets in adipocytes [4], [5], [6]. Under basal conditions, perilipin 1A may inhibit lipolysis by blocking lipase access to TAGs and/or by sequestering CGI-58 (also known as Abhd5) [7], while HSL is largely located in the cytoplasm. Agents that increase c-AMP activate c-AMP-dependent protein kinase (PKA) to phosphorylate perilipin 1A and HSL. Perilipin 1A phosphorylation releases CGI-58, which enables CGI-58 to activate adipose triglyceride lipase (ATGL). ATGL is the initiating lipase in lipolysis, as it removes a fatty acid moiety from TAG to form diacylglycerol. Additionally, phosphorylated HSL translocates from the cytoplasm to the lipid droplets where it interacts with phosphorylated perilipin 1A [8], [9]. HSL acts as the second lipase in the pathway, where it releases a fatty acid moiety from diacylglycerol to form monoacylglycerol. Monoacylglycerol is also further processed by monoacylglycerol lipase to form fatty acid and glycerol (not shown).

While it is clear that lipolysis is orchestrated by phosphorylation of several proteins, the specific relationship between phosphorylation events is not well understood. Murine perilipin 1A (National Center for Biotechnology Information (NCBI) reference sequence NP_783571.2) has six potential PKA phosphorylation sites [10] located at serines 81, 222, 276, 433, 492, and 517, referred to as PKA-sites 1–6, respectively [11]; splice variants of perilipin 1 (PLIN1b and PLIN1c), which lack PKA sites 4–6, are also less commonly expressed [5]. Human perilipin 1A (NCBI reference sequence NP_001138783.1) is similar to murine perilipin 1A, but lacks PKA-site 2. Furthermore, there are minor insertions and deletions between the amino acid sequences for perilipin 1A between the two species, making serines 497 and 522 of human perilipin 1A equivalent to serines 492 and 517 of murine perilipin 1A. PKA-sites 5 and 6 are likely to be critical for proper regulation of lipolysis. PKA-site 5 promotes agonist-induced lipid droplet dispersion [12], while PKA-site 6 maximizes activation of ATGL-dependent lipolysis [13]. The timing and extent of phosphorylation of perilipin 1A at these sites is unknown.

HSL is phosphorylated on serines 563, 659, and 660 by PKA at the initiation of lipolysis. Phosphorylation of HSL on serines 659 and 660 are activating, but the function of phosphorylation on serine 563 is unclear [14]. Additionally, HSL can be phosphorylated on serine 600 by extracellular signal-regulated kinase/mitogen-activated protein kinase (ERK-MAPK) which increases its activity [15], whereas phosphorylation of HSL on serine 565 by AMP-activated protein kinase, (AMPK) is inhibitory [14]. Recent work by Martin et al. [16] established the early time course and spatial distribution of HSL phosphorylation in murine 3T3L1 adipocytes, utilizing antibodies specific to HSL phosphorylation at either serine 563 (pHSL-serine 563) or serine 660 (pHSL-serine 660). Within a few seconds of exposure to FSK, pHSL-serine 660 appears near the plasma membrane, preceding translocation of HSL to lipid droplets. In contrast, pHSL-serine 563 appears with a delay relative to pHSL-serine 660 and is localized exclusively at the edges of lipid droplets. Collectively, it was proposed that separate pools of PKA, proximal to either the plasma membrane or lipid droplets, may orchestrate the differential phosphorylation of HSL at serine 660 and serine 563 [17].

The goal of the present study was to examine the specificity and time course of perilipin 1A phosphorylation at the initiation of lipolysis. Towards this end, monoclonal antibodies were developed which selectively label perilipin 1A phosphorylation at either PKA-site 5 (murine serine 492/human serine 497) or PKA-site 6 (murine serine 517/human serine 522). We utilized these reagents, in combination with high content analysis, to quantify the appearance of phospho-perilipin and phospho-HSL in adipocytes following exposure to lipolytic agents. We used FSK and isoproterenol, which increase cAMP, and L-γ-MSH, a peptide hormone that induces lipolysis by an incompletely characterized mechanism [18], [19], as model lipolytic agents. Our study reveals perilipin 1A is phosphorylated on PKA-site 5 in a manner similar to HSL-serine 660 (relatively rapid, and with equal efficacy by FSK and L-γ-MSH) whereas perilipin 1A PKA-site 6 is phosphorylated in a manner more similar to HSL-serine 563 (more slowly, with L-γ-MSH a stronger agonist than FSK). Comparison of the sequences surrounding the phosphorylation sites on perilipin and HSL suggests the presence of single or multiple arginine residues upstream from the phospho-acceptor serine may influence the differential phosphorylation of perilipin 1A and HSL at the initiation of lipolysis.

## Materials and Methods

### Ethics Statement

For certain experiments, human cells were utilized, which were obtained and de-identified by Zen-Bio, Inc, following written informed consent from the donors, utilizing a protocol approved by the Essex Institutional Review Board, Inc of Lebanon, New Jersey (“Surgical Waste Remnants” ZB02, 6/30/06, Amendment No. 1, 3/14/11; this protocol was most recently approved, June 13, 2011, and runs through May 28, 2013).

### Cell Culture

3T3L1 cells were obtained from ATCC and maintained in Growth Medium (Dulbecco's Modified Eagle's Medium supplemented with 10% fetal bovine serum, penicillin, streptomycin, amphotericin). Cells were passed a maximum of 17 times prior to use in experiments. To initiate adipogenesis, 3T3L1 cells were seeded in 75 cm^2^ flasks and allowed to grow to confluence. The cells were then exposed to Differentiation Media (Growth Medium additionally supplemented with 0.5 mM IBMX (3-Isobutyl-1-methylxanthine), 0.25 µM dexamethasone, and 1 µg/ml insulin). After 3 or 4 days in Differentiation Medium, the cells were harvested, seeded in 96-well glass bottomed dishes (Nunc Cat. No 73520-168, two 96-well dishes/flask) precoated with gelatin which was cross-linked with glutaraldehyde [20] at a density of 67,000 cells/well, and re-fed with Growth Medium. Experiments were performed 2 to 5 days after seeding into the 96-well dishes. With this protocol, a high degree of the cells typically featured clusters of lipid droplets. HeLa cells were also obtained from ATCC.

Human subcutaneous preadipocytes (a mixture of preadipocytes from 6 females, average age 39, average BMI = 0.273) were obtained via liposuction by Zen-Bio (Research Triangle Park, NC) and were plated (13,000 cells/well, passage 2) on glass-bottomed 96-well dishes precoated with gelatin that was cross linked with glutaraldehyde. To induce differentiation of the cells to adipocytes, the cells were exposed to Differentiation Medium (DM-2, Zen-Bio), which contains factors that induce adipogenesis in this cell type; alternatively, in certain experiments, the cells were exposed to a similar media, which featured the peroxisome proliferator-activated receptor gamma (PPARγ) agonist rosiglitazone (300 nM), which is also effective at inducing differentiation.

### Plasmids and Reagents

The cDNA sequence encoding human perilipin 1A was purchased from Origene (Rockville, MD). S497A and S522A mutations were introduced by using internal primers including the sequence encoding each serine. The codon for serine was altered to encode alanine. Subsequently, the wild-type or mutated perilipin 1A cDNAs were transferred to either GFP or mCherry vectors using appropriate restriction sites. Wild-type and mutated forms of perilipin 1A were sequenced to verify fidelity. Antibodies used to visualize HSL or perilipin 1A were from Vala Sciences Inc (San Diego, California). GP29, a guinea pig polyclonal antibody raised against perilipin, was from Progen (Heidelberg, Germany). 4,4-difluoro-1,3,5,7,8-pentamethyl-4-bora-3a,4a-diaza-s-indacene (equivalent to Bodipy 493/503™ Life Technologies) was used to visualize lipid droplets. Lipofectamine 2000, AlexaFluor 647-anti mouse secondary antibody, Cell Mask Blue, HCS LipidTox DeepRed, and SlowFade Gold mounting solution were from Life Technologies (Carlsbad, California). HiLyte Fluor™ 647 goat-anti-rabbit (far-red fluorescent secondary antibody) was from AnaSpec (Fremont, California). Texas Red conjugated goat-anti-mouse (red fluorescent secondary antibody) was from Jackson ImmunoResearch Laboratories.

### Production of Mouse Monoclonal Antibodies

Mouse monoclonal antibodies were developed and characterized by the Monoclonal Antibody Core Facility at Baylor College of Medicine. Mice were inoculated with peptides containing amino acid sequences identical to those surrounding PKA-site 5 or PKA-site 6 of the human perilipin 1A sequence (Figure 1). N-α-Fmoc-O-benzyl-L-phosphoserine was substituted for serine at the phospho-acceptor site, to create a stable analogue of phospho-serine. Hybridomas were prepared from sera-positive mice by previously described techniques [21], [22], [23] and verified for the production of antibodies against the immunizing peptide by ELISA. The antibodies developed in this study, designated anti-pPeri-site 5 and anti-pPeri-site 6, are available for purchase from Vala Sciences Inc (catalogue items MAb #4855 and MAb #4856, respectively).

### Characterization of Antibody Specificity

After isolation of hybridomas secreting monoclonal antibodies, specificity to codon mutation was tested using wild-type and mutant perilipin constructs in GFP and mCherry vectors, respectively. HeLa cells were plated in 6 well plates 24 h prior to transfection. HeLa cells were transfected using Lipofectamine 2000 with a single plasmid (either GFP-*PLIN1* (wild type), mCh-*PLIN 1* S497A, or mCh-*PLIN1* S522A) used per transfection reaction. The next day, the cells were detached and seeded on 22 mm poly-D-lysine coated coverslips placed in 24-well dishes. Approximately 8 hr later, oleic acid complexed to fatty acid-free BSA (a kind gift from Larry Chan, Baylor College of Medicine, 550 µM) was added to induce lipid droplet formation. After an overnight incubation with oleic acid, the cells were treated with forskolin (24 µM) for 7 min to increase PKA activity. Treatments and transfections were performed in OPTI-MEM (Invitrogen).

Following treatment with forskolin or DMSO, cells were washed and fixed in 4% paraformaldehyde (ultrapure, Electron Microscopy Sciences, Hatfield, PA) for 30 minutes at room temperature. Coverslips were quenched with 100 mM ammonium chloride and washed three times with PIPES/HEPES/EGTA/MgCl2 (PEM) buffer, prepared at a final pH of 6.8. Cells were permeabilized with 0.2% Triton X-100 in PEM for 10 minutes, washed three times with PEM and blocked in 5% milk in PEM/0.01% saponin blocking solution for 30 minutes at room temperature. Antibodies were then diluted in the blocking solution and incubated overnight at 4°C. Subsequently, coverslips were washed with PEM and incubated with AlexaFluor 647-conjugated anti-mouse secondary antibodies (Life Technologies) for 1 h at room temperature. Cells were again washed 3 times and incubated with CellMask Blue (1 µg/ml), and DAPI (10 µg/ml) in PEM for 45 minutes at room temperature. Dyes were then aspirated, PBS/0.01% azide added, and cells imaged immediately. Coverslips were mounted with SlowFade Gold (Invitrogen). Images were acquired with a Beckman coulter IC-100 Image Cytometer equipped with a Nikon S Fluor 20X/0.75NA objective or a DeltaVision Core Image Restoration Microscope (Applied Precision, Issaquah, WA) using a 60X, 1.42 NA Plan Achromat objective (Olympus, Center Valley, PA), and a Photometrics CoolSnap HQ2 CCD camera.

### siRNA Transfection

Preadipocytes were obtained from Zen Bio (SP-F-SL, lot# SL0048) and were plated directly on 96 well plate at the density of 15000 cells per well. siRNA transfection was performed at the time of plating. A mix of the cells, media (DMEM, Gibco 11966-25 500 ml, lot# 1095492), siRNA (Silencer® Select, Life Technologies), and transfection reagent (RNAI max, Invitrogen, 13778-075, lot# 804296) was prepared, and 200 µl of the mixture was added per well. The sense sequence of the perilipin 1A siRNA was GCACAUACCCUGCAGAAGA (5′ to 3′); additionally, an siRNA that does not correspond to a human gene was utilized as a control. The perilipin 1A and control siRNAs were tested at four different concentrations: 0 nM, 10 nM, 20 nM, and 50 nM. 48 hours post transfection, transfection media was removed, and differentiation medium was added for 6 days. Cells were then treated with forskolin at a final concentration of 6 µM in DMEM for 30 minutes, then fixed and labeled for nuclei, lipid droplets, and primary antibodies (anti-pPeri-ser 5, anti-pPeri-ser6, and GP29) via methods described below.

### Immunofluorescence

Cells were plated on 96-well dishes and exposed to experimental treatments, after which they were fixed with 4% paraformaldehyde plus 0.025% glutaraldehyde. The cells were then permeabilized with 0.1% Triton-X-100 (20 minutes) and blocked for 60 minutes in blocking buffer (10% goat serum, 3% BSA, 0.02% sodium azide in PBS). Fixed cells were labeled with primary antibodies diluted in blocking buffer for either 60 minutes at 37 C with rotation, or overnight at 4 C. The samples were then rinsed 3X with PBS, incubated for one hour with appropriate secondary antibodies (ether goat- anti-rabbit, goat- anti-mouse, or goat-anti- guinea pig) conjugated to red channel or far-red channel fluorophores, along with the Lipid Droplet Stain to label lipid droplets in the green channel. Following incubation with the secondary reagents, the plates were rinsed 3X with PBS. DAPI was then added; the dishes then sealed and incubated a further 20 minutes at room temperature, prior to automated image acquisition. In experiments in which peptides were tested for their ability to block primary antibody binding, the primary antibodies were preincubated with 0 to 10 µg peptide in a volume of 300 µl for 30 minutes at 37 C.

### Conventional Fluorescence Microscopy

Images were acquired with a Beckman Coulter IC 100 using established methods [20]. This instrument includes a Nikon Eclipse microscope featuring an automated stage interfaced to a fluorescence light source and filter wheel and cubes with filters for DAPI (excitation = 350±25 nm, emission = 465±25 nm) green channel (excitation = 490±10 nm, emission = 535±15 nm), red channel (excitation = 555±12.5 nm, emission = 615±26 nm), and far-red (excitation = 645±15 nm, emission = 715±36 nm) channel fluorescence. The workstation includes a Windows computer, which controls stage positioning, and data acquisition. Images were acquired with a Hamamatsu Orca ERG progressive scan 1344×1024 cooled interline CCD camera, utilizing 2 x 2 binning. Typically, 4 images (representing a 2 x 2 contiguous image set) were acquired in the center of each well with either a 20×0.5 NA (resulting in 0.6848×0.6848 µm2//pixel) or a 40×0.75 NA “dry” objective (resulting in 0.344×0.344 µm2/pixel). Images were stored as gray-scale bit mapped images (*.bmp).

### Confocal Microscopy

An LSM 710 NLO Zeiss Multiphoton Laser Point Scanning Confocal Microscope was used to scan adipocytes labeled with newly developed phospho-perilipin 1A specific antibodies, and a reference antibody to perilipin 1A. For these scans, a multi-photon Mai-Tai DeepSee HP laser was used to excite DAPI with 728 nm (pinhole = 601 µm); single channel lasers at 514 (31 µm), 561 (36 µm), and 622 (42 µm) were used for image acquisitions in the green, red, and far-red fluorescence channels, respectively. 20×0.8 NA M27 Plan apochromat and 63×1.4 NA M27 DIC objectives were used.

### High Content Analysis

CyteSeer® (Vala Sciences Inc) is a Java-based PC/Mac/Linux- compatible cell image analysis program designed specifically for high content analysis. CyteSeer®’s Colocalization Algorithm has been specifically developed for the analysis of lipid droplets and lipid droplet-associated proteins [20], [24], [25]. The Colocalization Algorithm utilizes the nuclear image from each field of view to identify the nuclei and to estimate cellular boundaries; this information is then applied to the lipid and protein images, to estimate the level of expression of lipid droplets and protein for each cell. In this study, the data parameter Area Pm (area of the protein mask), derived by the Colocalization Algorithm of CyteSeer® is presented. Area Pm corresponds to the area within each cell identified by the algorithm as being positive for the expression of protein (µm^2^/cell). A second data parameter, Tii Pi Pm, is reported for certain experiments in which the effect of blocking peptides were tested for their ability to reduce the labeling intensity obtained with the primary antibodies. Tii Pi Pm is the “Total integrated intensity” (sum of intensities of the pixels), in the “Protein image” (the image channel representing either perilipin 1A or HSL), in the “Protein mask” (the pixels within the cell, identified by CyteSeer® as being positive for protein expression). Thus, Tii Pi Pm reports the overall intensity of the cell for the protein that is labeled [20]. In experiments in which perilipin 1A and HSL were covisualized, the images were analyzed twice, first for perilipin 1A, and second for HSL.

### Western Blotting

3T3L1 cells were plated and differentiated in 6 well plates. After stimulation, cells in each well were rinsed with ice-cold PBS and lysed in 75 µl buffer containing 50 mM Tris, pH 7.4, 100 mM NaCl, 1% sodium deoxycholate, 4% Nonidet P-40, 0.4% SDS, proteases and phosphatase inhibitors (Sigma). Lysates were incubated on ice for 15 min, frozen overnight at −80°C, thawed on ice and centrifuged at 13,000 rpm. The resulting supernatants were stored at –80°C, and protein concentrations were determined using the BCA kit (Pierce, Rockford, IL). Next, 5–40 µg of lysate were loaded on 12% BisTris gel, subjected to electrophoresis and transferred to BiotraceTM PVDF membrane (Pall, New York). After blocking with 5% milk protein in TBST for 1 hour, membranes were incubated overnight with primary antibodies. Primary antibodies were detected using an HRP-conjugated secondary antibody and visualized by ECL.

### Statistical Analysis

GraphPad Prizm was used to test for statistically significant differences between controls and experimental groups (ANOVA followed by Dunnett’s test, or ANOVA followed by Tukey's test).

## Results

### Generation of Monoclonal Antibodies Specific for Phosphorylated Forms of Perilipin 1A

To generate novel monoclonal antibodies directed to phosphorylated forms of perilipin 1A, peptides analogous to the amino acid sequences surrounding serine 497 (PKA-site 5) or serine 522 (PKA-site 6) of human perilipin 1A were synthesized. The serines of these peptides were substituted with a chemical moiety (see Materials and Methods), which acts as a mimetic for phospho-serine. Mice were injected with the peptides, and sera-positive animals were screened for MAb-secreting hybridomas. Aliquots of media from the hybridomas were tested against the inoculating peptides via ELISA. Clones that scored positive for recognition of the inoculating peptides by ELISA were evaluated for their ability to produce antibodies that visually label adipocytes pretreated with forskolin in a pattern consistent with perilipin 1A. We selected two monoclonal antibodies, designated anti-pPeri-site 5 and anti-pPeri-site 6, that showed strong ELISA reactions and appropriate labeling patterns.

### Validation of the Specificity of the Antibodies

To evaluate the specificity of the anti-pPeri-site 5 and anti-pPeri-site 6 antibodies, perilipin 1A mutant plasmids were utilized, in which alanine was substituted for either serine 497 (to remove PKA-site 5) or serine 522 (to remove PKA-site 6). The coding region of each perilipin 1A mutant construct was fused to mCherry, a red channel fluorescent protein, to create mCh-perilipin S497A and mCh-perilipin S522A. Separate HeLa cell preparations were transiently transfected with GFP-perilipin 1A (wt), mCh-perilipin 1A (S497A), or mCh-perilipin 1A (S522A). The cells were then exposed to oleic acid to promote formation of lipid droplets for 24 h. Subsequently, FSK+IBMX was used to activate PKA. Cells were then fixed, stained for nuclei, and labeled with either anti-pPeri-site 5 or anti-pPeri-site 6. Antibody labeling was visualized with secondary anti-mouse antibodies conjugated to a far-red fluorophore.

**Figure 2:**
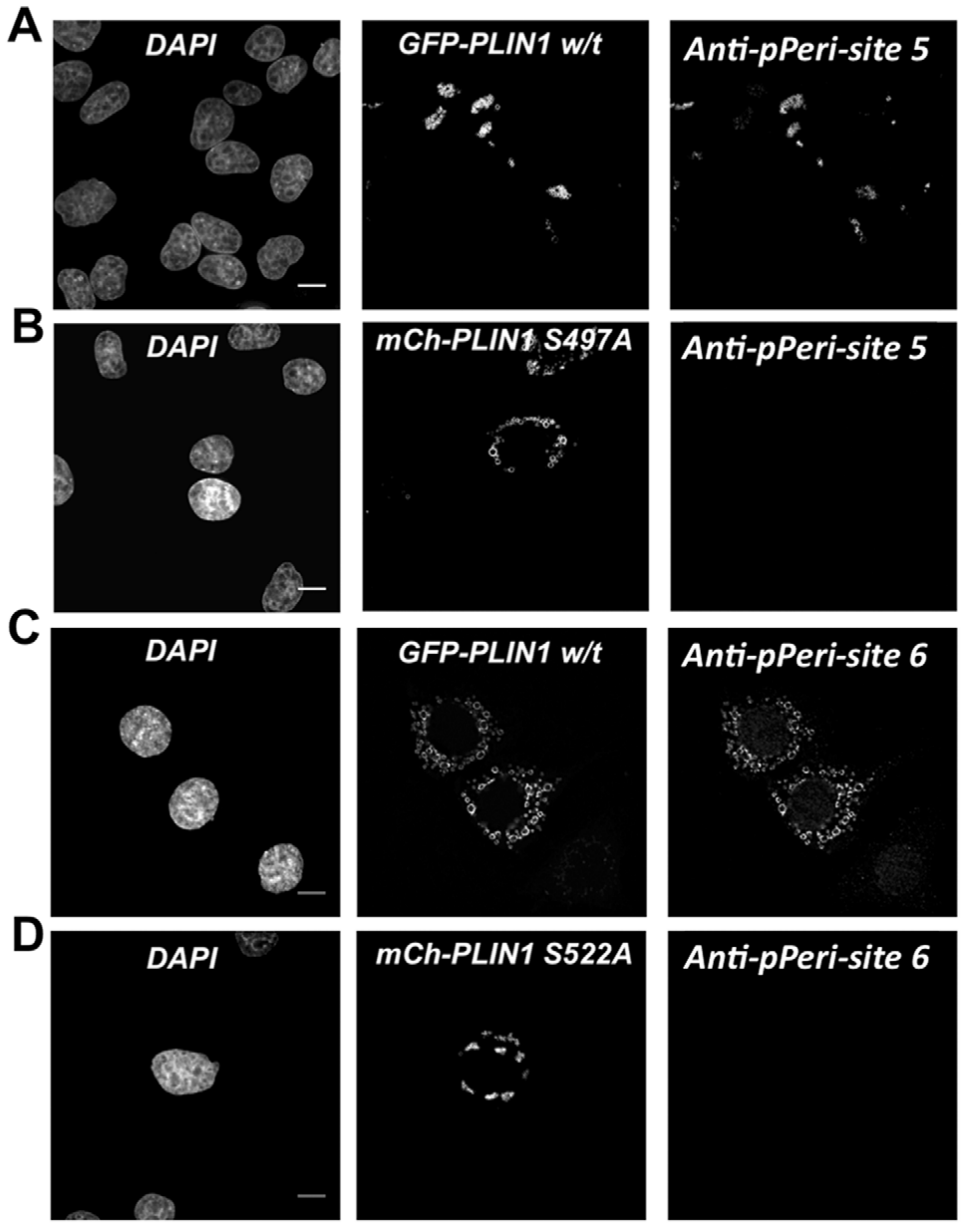
Determination of antibody specificity for perilipin 1A PKA-site 5, and PKA-site 6. Hela cells were transfected separately with plasmids encoding wild-type (GFP, green) or mutant (mCherry, red) perilipin 1A (*PLIN1*) plasmids. Oleic acid was added for 24 h, followed by 7 min treatment with 24 µM forskolin plus 125 µM IBMX and the cells labeled with either anti-pPeri-site 5 or anti-pPeri-site 6. For each condition, images are shown for nuclei (DAPI), fluorescent protein (either GFP or m-Cherry), or for antibody labeling (the far red fluorescence channel). A, Cells expressing GFP-w/t-perilipin 1A labeled with anti-pPeri-site 5. B, Cells expressing mCh-perilipin 1A S497A, which did not label for anti-pPeri-site 5. C, Cells expressing GFP-w/t-perilipin 1A labeled with anti-pPeri-site 6. D, Cells expressing mCh-perilipin 1A S522A, which did not label with anti-pPeri-site 6.

For cells prepared in this manner, the anti-pPeri-site 5 antibody labeled cells expressing wild type perilipin 1A (Figure 2A), but did not label cells expressing perilipin 1A with the S497A mutation (Figure 2B). Similarly, the anti-pPeri-site 6 antibody also labeled cells expressing wild type perilipin 1A (Figure 2C), but did not label cells expressing perilipin 1A with the S522A mutation (Figure 2D). These experiments demonstrate that specific labeling of perilipin 1A by anti-pPeri-site 5 and anti-pPeri-site 6 requires serine phosphorylation at position 497 and 522, respectively.

Guinea pig polyclonal antibody #GP29, raised against the N-terminus of perilipin 1A, is widely used to label human [20] and mouse 3T3L1 adipocytes [26]. To further validate the labeling of perilipin 1A by the anti-pPeri-site 5 and anti-pPeri-site 6 antibodies, 3T3L1 adipocytes were exposed to either 6 µM FSK or 100 nM L-γ-MSH for 20 minutes, then fixed, permeabilized, and colabeled with either anti-pPeri-site 5 plus GP29, or with anti-pPeri-site 6 plus GP29. This was possible since the murine and guinea pig antibodies are from different host animals, and can thus be specifically visualized with different secondary antibodies and fluorophores.

**Figure 3:**
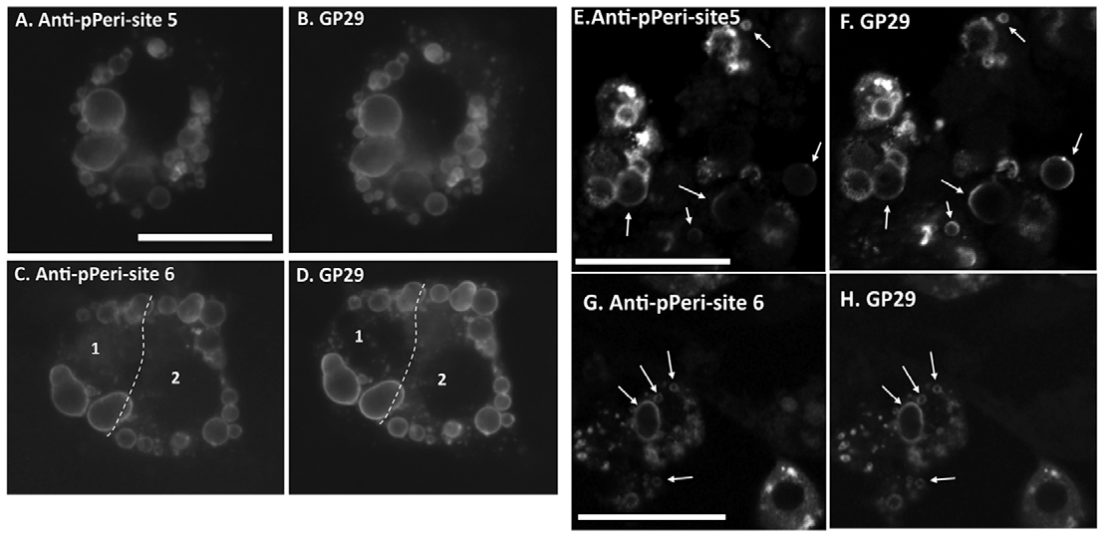
Labeling of adipocytes with anti-pPeri-site 5, anti-pPeri-site 6, and GP29. 3T3L1 adipocytes were exposed to agonists (either 6 µM FSK or 100 nM L-γ-MSH) for 20 minutes, then fixed, and labeled with either anti-pPeri-site 5 or anti-pPeri-site 6 and GP29. *Conventional microscopy (A–D)*. Alexa Fluor® 488 goat-anti-mouse and Alexa Fluor® 647 goat anti-guinea pig secondary antibodies were used to visualize the mouse monoclonal and guinea pig polyclonal antibodies, respectively. A, A FSK-treated cell visualized for anti-pPeri-site 5. B, The same cell, visualized for GP29. C, A cluster of two FSK-treated cells (labeled 1 and 2) visualized for anti-pPeri site 6; the dashed line is the boundary between the cells as estimated by CyteSeer®. D, The same cell cluster, visualized for GP29. Images were acquired with a 40×0.75 NA objective. E–H, *Confocal microscopy.* Alexa Fluor® 568 goat-anti-mouse and Alexa Fluor® 647 goat anti-guinea pig secondary antibodies were used to visualize the mouse monoclonal and guinea pig polyclonal antibody labels, respectively. E, FSK-treated cells visualized for anti-pPeri-site 5. F, The same cells visualized for GP29. Images were acquired with a 20×0.8 NA objective. G, L-γ-MSH-treated cells visualized for anti-pPeri-site 6. H, The same cells visualized for GP29. Images were acquired with a 63×1.4 NA objective. Examples of prominent labeling at the edges of lipid droplets are indicated by arrows (lipid droplets, which were visualized in a separate channel, are not shown). Scale bars are 50 µm.

For images acquired from these FSK-treated adipocytes, via conventional microscopy, the labeling by anti-pPeri-site 5 was virtually identical to the labeling pattern obtained with GP29 (Figure 3A vs. 3B). Furthermore, virtually identical labeling patterns were also seen for anti-pPeri-site 6 compared to GP29 (Figure 3C vs. 3D). Indeed, Pearsons’ Correlation Coefficients, calculated on a cell by cell basis by CyteSeer®, yielded a value of 0.96 for the cell depicted in Figure 3A and 3B. For 3C and 3D, there are two juxtaposed cells (note the two dark central regions in 3D, which correspond to the location of separate nuclei), for which CyteSeer® calculated Pr values of 0.88 (cell 1) and 0.94 (cell 2), respectively. P_r_ has a theoretical range from −1.0 (perfect exclusion) to +1.0 (perfect coincidence). Thus, the observed P_r_ values strongly support the observation from visual inspection that distribution of phospho-perilipin 1A labeled by the monoclonal antibodies coincides with the distribution of total perilipin 1A labeled by GP29. For all of the antibodies, labeling was specific for the edges of lipid droplets, which is the known intracellular location of perilipin 1A. Virtually identical images for anti-pPeri-site 5 and GP29 and for anti-pPeri-site 6 and GP29 were also obtained by confocal microscopy (Figure 3E to 3H), confirming the labeling at the edges of the lipid droplets by both anti-pPeri-site 5 and anti-pPeri-site 6, and the coincidence of the labeling with GP29.

**Figure 4:**
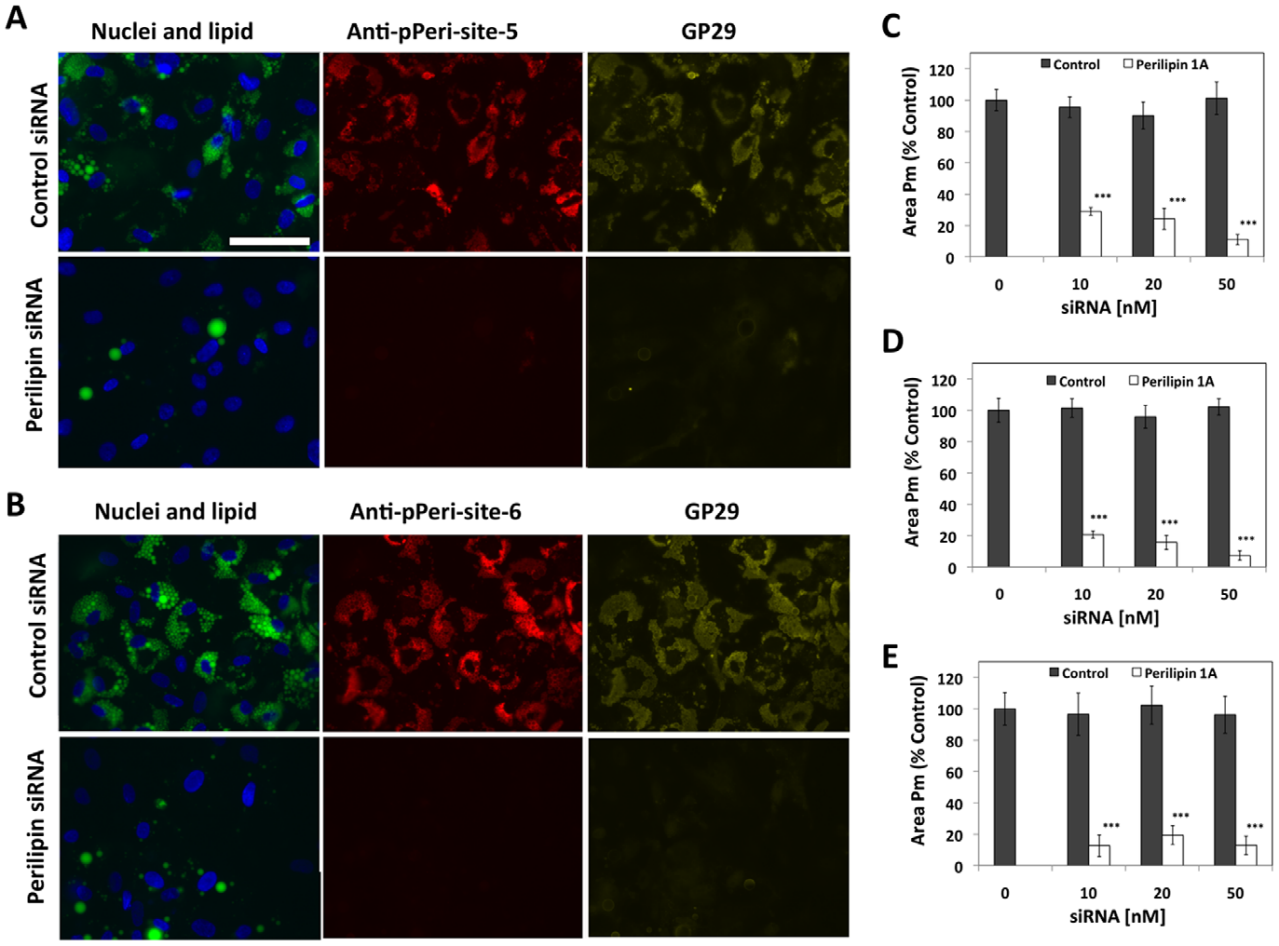
Downregulation of anti-pPeri-site 5, anti-pPeri-site 6, and GP29 labeling by siRNA to perilipin 1A. Preadipocytes were transfected with either control or perilipin 1A siRNA (0 to 50 nM), exposed to differentiation medium for 6 days, treated with 6 µM FSK for 20 minutes, then fixed and labeled for nuclei (blue), lipid (green), and either anti-pPeri-site 5 or anti-pPeri-site 6 (red) plus GP29 (yellow). A, Representative fields of view are shown for cells transfected with 10 nM siRNA and labeled with anti-pPeri-site 5 plus GP29. B, Representative fields of view are shown for cells transfected with 10 nM siRNA and labeled with anti-pPeri-site 6 plus GP29. C, D, and E are mean values for Area of the Protein mask (Area Pm), for GP29, anti-pPeri-site 5, and anti-pPeri-site 6, respectively. Data are normalized to the 0 siRNA control, and each bar represents the mean ± SD, for n = 6 wells. *** p<0.001 for perilipin vs. control siRNA at each concentration (Student’s t-test).

In a further experiment to test the specificity of the antibodies, human preadipocytes were transfected with either control siRNA (scrambled sequence) or an siRNA corresponding to human perilipin 1A, at concentrations ranging from 0 to 50 nM. Following transfection, the cells were exposed to Differentiation Medium for 6 days, treated with 6 µM FSK for 20 minutes, and fixed and labeled for nuclei, lipid droplets, and for both phospho-perilipin (utilizing either anti-pPeri-site 5 or anti-pPeri-site 6) and total perilipin (utilizing GP29). For cells transfected with 10 nM control siRNA, most of the cells featured numerous lipid droplets and were strongly labeled by GP29, anti-pPeri-site 5, and anti-pPeri-site 6 (Figure 4A and 4B). In contrast, cells transfected with siRNA to perilipin 1A featured much weaker labeling by anti-pPeri-site-5, anti-pPeri-site6, and GP29. Interestingly, the cells transfected with the perilipin siRNA also featured fewer lipid droplets. This is consistent with expectations, as knockout of perilipin 1A in mice results in a leaner phenotype [27], [28] and siRNA to perilipin also downregulates lipid droplets in a murine adipocyte cell model [29]. The potential explanation for this is that reduction of perilipin expression in adipocytes likely leads to increased basal lipolysis and reduction of triglyceride stores.

To quantify the effects of the siRNAs, images from the experiment were analyzed with CyteSeer®. Area Pm values were not affected by the control siRNA, but the perilipin siRNA reduced labeling by GP29, anti-pPeri-site5, and anti-pPeri-site 6 in a very similar fashion, up to 90% (Figure 4C–E).

The above results confirm that anti-pPeri-site 5 labels perilipin 1A which has been phosphorylated at PKA-site 5, whereas anti-pPeri-site 6 labels perilipin 1A, which has been phosphorylated at PKA-site 6. Note that for the purposes of quantification, perilipin 1A labeled by the anti-phospho-perilipin 1A antibodies, will be referred to as pPeri-site 5 and pPeri-site 6, respectively.

### Labeling of Endogenous Phospho-perilipin 1A in Human Adipocytes at the Initiation of Lipolysis

**Figure 5:**
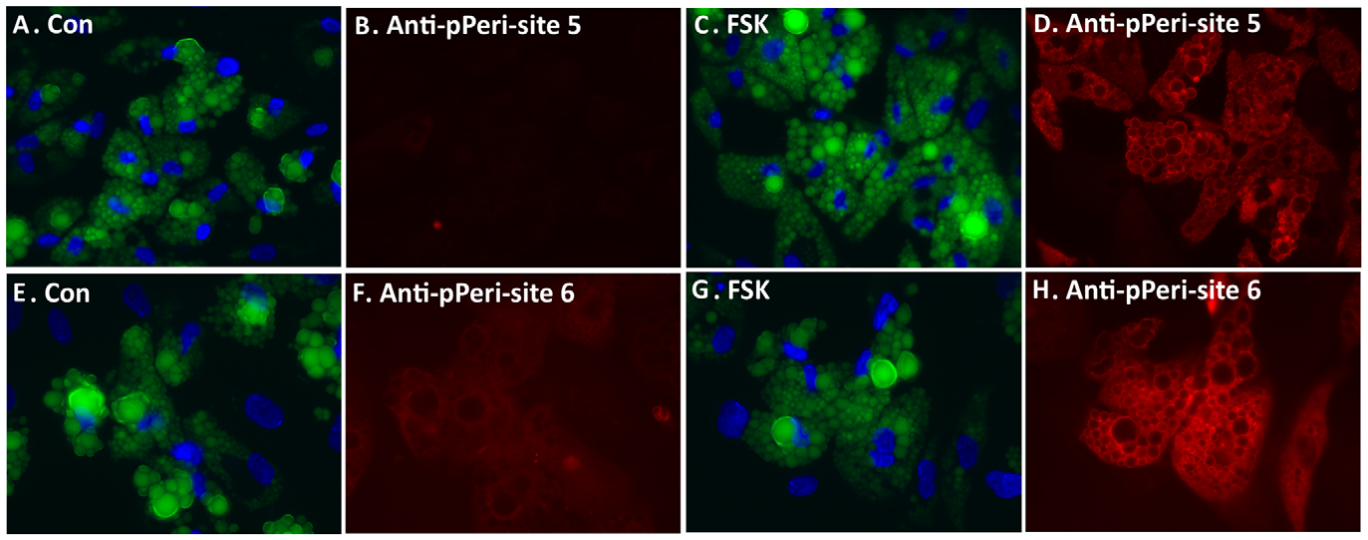
Labeling of FSK-treated human adipocytes with anti-pPeri-site 5 and anti-pPeri-site 6 antibodies. Human subcutaneous adipocytes were exposed to either control medium, or medium supplemented with 10 µM FSK and 500 µM IBMX for 2 minutes, then fixed and labeled for nuclei, lipid droplets, and with either anti-pPeri-site 5 or anti-pPeri-site 6. A goat-anti-mouse secondary antibody coupled to Texas Red was used to visualize the phospho-perilipin antibodies in the red fluorescent channel. A, Control adipocytes are shown visualized for nuclei (blue) and lipid droplets (green). B, the same field is shown visualized for anti-pPeri-site 5 (red). C, FSK/IBMX-treated adipocytes are shown visualized for nuclei and lipid droplets. D, The same field is shown visualized for anti-pPeri-site 5. E, Control adipocytes are shown visualized for nuclei and lipid droplets. F, The same field is shown visualized for anti-pPeri-site 6. G, FSK/IBMX-treated adipocytes are shown visualized for nuclei and lipid droplets. H, The same field is shown, visualized for anti-pPeri-site 6. Scale bar = 50 µm.

Next, experiments were conducted to test the ability of these antibodies to recognize endogenous phospho-perilipin 1A in adipocytes under lipolytic treatments. Human adipocytes exposed to control medium showed little labeling for with the anti-pPeri-site 5 antibody (Figure 5A and 5B). In contrast, following exposure to FSK/IBMX, labeling by anti-pPeri-site 5 was very strongly increased (Figure 5C and 5D), suggesting FSK/IBMX strongly increases phosphorylation of perilipin 1A at PKA-site 5. Similarly, for anti-pPeri-site 6, there was minimal labeling of human adipocytes under basal conditions (Figure 5E and 5F), and prominent labeling following exposure to FSK/IBMX (Figure 5G and 5H), suggesting that FSK/IBMX also leads to phosphorylation of perilipin 1A PKA-site 6.

### Differential Phosphorylation of Perilipin 1A in Response to L-γ-MSH Versus FSK

**Figure 6:**
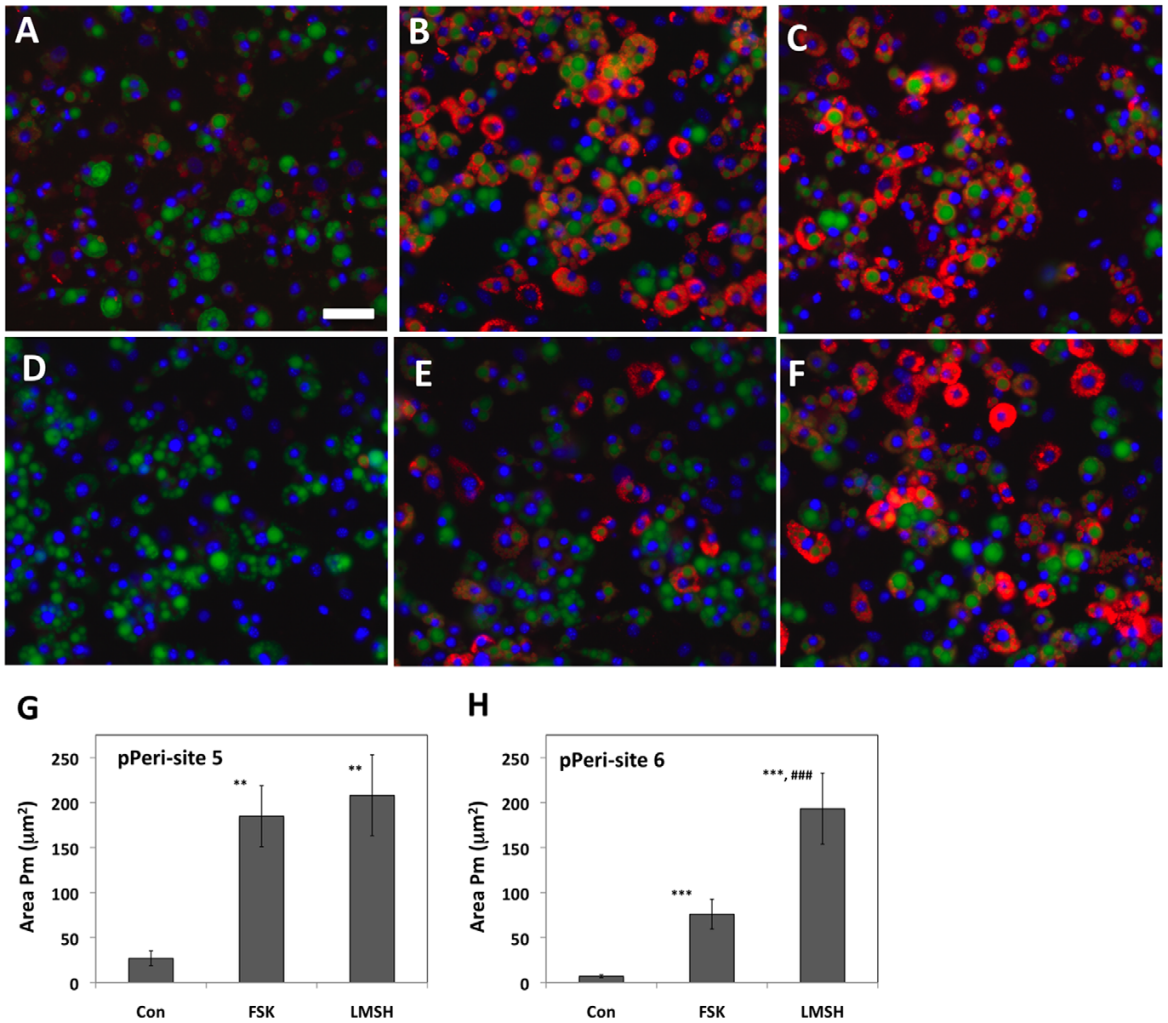
Differential phosphorylation of perilipin 1A by forskolin (FSK) and LγMSH. 3T3L1 adipocytes were exposed to either FSK (6 µM) or L-γ-MSH (100 nM) for 5 minutes, then fixed and labeled for nuclei (blue), lipid droplets (green), and phosphorylated perilipin (red). The cells were then imaged (20Xobjective, 4 images/well) and the images analyzed utilizing the Colocalization algorithm. A, B, and C, Images are shown of adipocytes exposed to control, FSK, or LγMSH, respectively, labeled with anti-pPeri-site 5. D, E, and F, Images are shown of adipocytes exposed to control, FSK, LγMSH, respectively, labeled with anti-pPeri-site 6. G and H represent Area Pm for pPeri-site 5 and for pPeri-site 6, respectively. Each bar represents the mean SD for n = 3 wells/condition (an average of 532 cells/well) for G, or n = 8 well/condition (an average of 272 cells/well) for H. ** p<0.01 vs. Con (ANOVA followed by Tukey’s test). *** P<0.001 vs. Con. ^###^ p<0.001 vs. FSK. Scale bar = 50 µm.

We [25] and others [18], [19] have examined the phosphorylation of HSL in 3T3L1 adipocytes in response to FSK and L-γ-MSH. To examine the phosphorylation of perilipin 1A in response to these agents, murine 3T3L1 adipocytes were exposed to either FSK or L-γ-MSH for 5 minutes, then fixed, and labeled for nuclei and lipid droplets, along with either anti-pPeri-site 5 or anti-pPeri-site 6. 3T3L1 adipocytes exposed to control medium exhibited relatively little labeling with anti-pPeri-site 5 (Figure 6A). Treating the cells with FSK (6 µM) or L-γ-MSH (100 nM) led to strong and similar increases in labeling for anti-pPeri-site 5 (Figure 6B and 6C). Interestingly, the results obtained with the anti-pPeri-site 6 antibody were qualitatively distinct from the results for anti-pPeri-site 5. For the anti-pPeri-site 6 antibody, labeling was dim for control cells (Figure 6D) and increased by both FSK (Figure 6E) and L-γ-MSH (Figure 6F); however, the effect of L-γ-MSH was stronger than the effect of FSK.

To quantify the agonist-induced appearances of pPeri-site 5 and pPeri-site 6, images from the experiment were analyzed with CyteSeer® utilizing the Colocalization Algorithm. For pPeri-site 5, Area Pm (the area of pixels that are positive for pPeri-site 5 on a per cell basis) averaged 27 µm^2^/cell for control cells (Figure 6G), suggesting a small but measurable degree of basal phosphorylation of perilipin 1A at PKA-site 5. Exposure to FSK or L-γ-MSH led to 7-fold and 8-fold increases in Area Pm, respectively. These responses were significantly different compared to control wells (p<0.01), but were not significantly different from each other. For pPeri-site 6, Area Pm values for controls averaged 7 µm^2^/cell (Figure 6H). Exposure to FSK or L-γ-MSH led to 11-fold and 27-fold increases in Area Pm, respectively. Statistically, these responses were different both from the controls and from each other at a high level of significance (p<0.001). Thus, in this experiment, L-γ-MSH was nearly 3-fold more effective than FSK at inducing the appearance of pPeri-site 6.

### Concurrent Analysis of Phospho-perilipin 1A and Phospho-HSL in 3T3L1 Adipocytes Subjected to FSK or L-γ-MSH

In the experiment described above, FSK and L-γ-MSH were equally effective at inducing the appearance of pPeri-site 5, but L-γ-MSH was a stronger agonist at inducing the appearance of pPeri-site 6. This prompted us to ask whether or not these agents might also lead to differential phosphorylation of HSL. Accordingly, a time course experiment was conducted in which 3T3L1 adipocytes were exposed to control medium, 6 µM FSK, or 200 nM L-γ-MSH, for time periods of 1, 5, or 20 minutes. The cells were then fixed as before, but co-labeled for both phospho-perilipin 1A and phospho-HSL. This was possible since the anti-perilipin 1A antibodies were raised in mice, whereas the anti HSL antibodies were raised in rabbit, and thus these antibodies are immunologically distinct. In the experiments shown, adipocytes were co-labeled with anti-pPeri-site 5 plus anti-pHSL-serine 563 antibodies, or co-labeled for with anti-pPeri-site 6 plus anti-pHSL-serine 660 antibodies. Following labeling, the cells were visualized in 4 fluorescent channels and the images analyzed with the CyteSeer® Colocalization algorithm.

**Figure 7:**
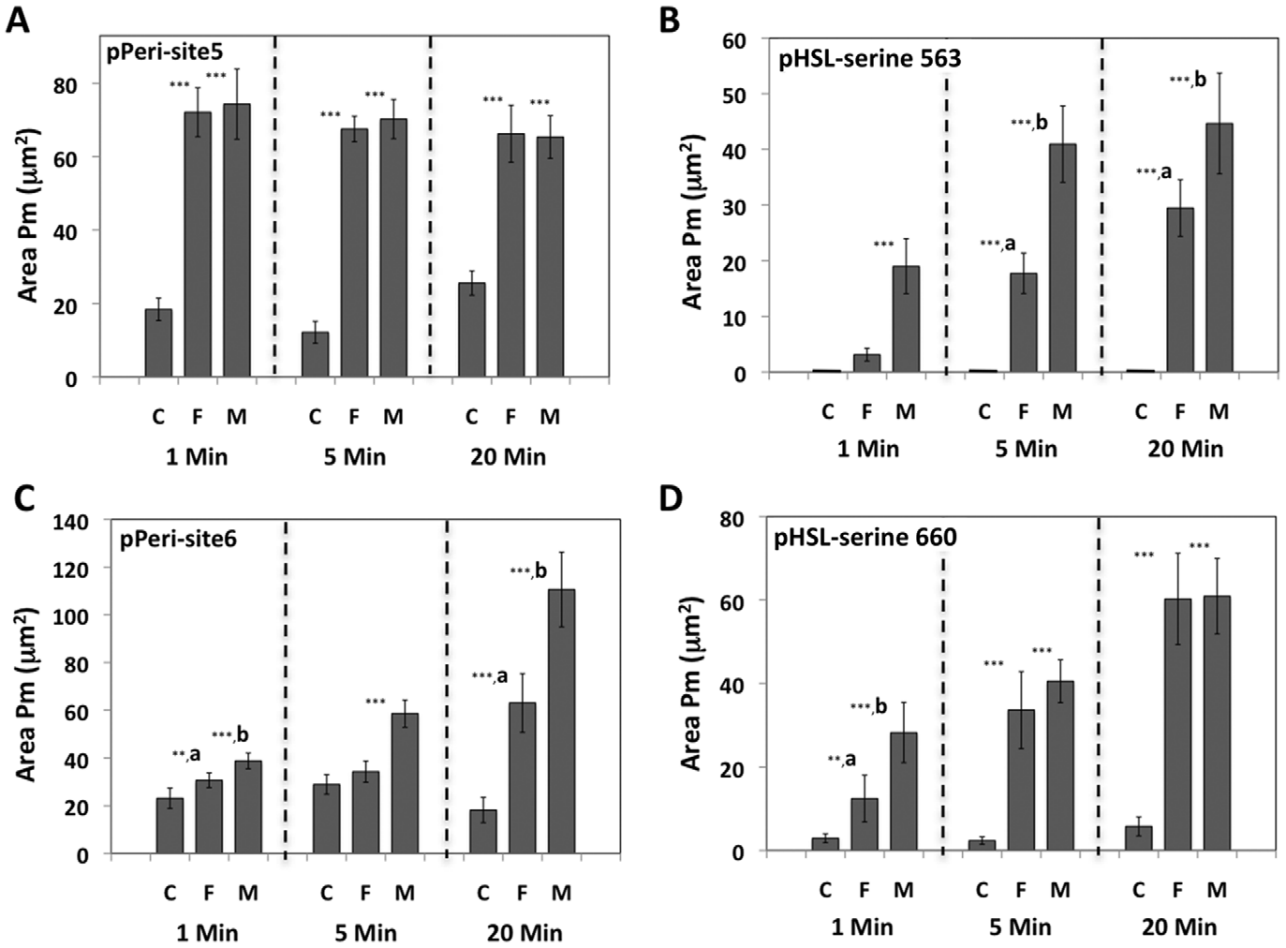
Phosphorylation of Perilipin 1A and HSL in response to FSK and L-γ-MSH. 3T3L1 adipocytes were exposed to control medium, 6 µM forskolin (F), or 200 nM L-γ-MSH (M) for 1, 5, or 20 minutes, then fixed and co-labeled with either anti-pPeri-site 5+ anti-pHSL-serine 563, or anti-pPeri-site 6+ anti-pHSL-serine 660. Images were obtained with a 20X objective (4 images/well, representing an average of 930 cells/well). A and B represent Area Pm values obtained for pPeri-site 5 and pHSL-serine 563. C and D represent Area Pm values obtained for pPeri-site 6 and pHSL-serine 660, respectively. Each bar represents the mean ± SD for n = 7 to 8 wells. ** p<0.01 compared to controls (ANOVA followed by Tukey’s test); *** p<0.001 compared to controls. ^a^ p<0.05 vs. controls and M; ^b^ p<0.05 vs. controls and F.

For pPeri-site 5 (Figure 7A), Area Pm values averaged about 20 µm^2^ for control cells. Exposure to either FSK or L-γ-MSH led to approximately 4-fold increases in Area Pm which was immediate and sustained throughout the time course. Thus, phosphorylation of perilipin 1A at PKA-site 5 occurs rapidly for both agonists, and, FSK and L-γ-MSH were equally effective.

For pHSL-serine 563 (Figure 7B), Area Pm values for controls were negligible. For cells exposed to FSK for 1 minute, Area Pm for FSK was approx. 4 µm^2^ whereas Area Pm for L-γ-MSH averaged about 20 µm^2^, which was significantly increased relative to the controls. At the 5 minute time point, Area Pm values averaged approx. 19 µm^2^ for FSK and about 41 µm^2^ for L-γ-MSH and these values were statistically different from controls. At the 20 minute time point, the data for FSK and L-γ-MSH were somewhat higher than for the 5 minute time point. At all time points, the differences between the L-γ-MSH and FSK were statistically significant.

For pPeri-site 6 (Figure 7C) at the one minute time point, Area Pm values averaged about 22 µm^2^ for control samples, and FSK and L-γ-MSH increased Area Pm by approximate 20% and 50%, respectively. At the 5 minute time point, the response for L-γ-MSH was 2-fold greater than the basal level of Area Pm, while the effect of FSK was not significantly different from control cells. At the 20 minute time point, FSK and L-γ-MSH increased Area Pm by approximately 3-fold and 5-fold, respectively. At all time points, the data values obtained with L-γ-MSH were significantly elevated compared to the data for FSK.

For pHSL-serine 660 (Figure 7D), Area Pm values averaged about 5 µm^2^ for control cells at the 1 minute time point, whereas FSK and L-γ-MSH increased Area Pm by approximately 4-fold and 9-fold, respectively. At the 5 minute time point, FSK and L-γ-MSH elicited similar responses and exhibited levels approximately 10-fold above the control values. At the 20 minute time point, both agents elicited very strong, virtually identical responses.

It is worth emphasizing that the data for Figure 7A and 7B were obtained from the same wells on the 96-well dish. Thus, FSK and L-γ-MSH elicited the appearance of pPeri-site 5 and pHSL-serine 563 with different kinetics and agonist efficacies within the same samples. Similarly, the data for Figure 7C and 7D were also obtained from the same wells on the 96-well dish, so FSK and L-γ-MSH also elicited the appearance of pPeri-site 6 and pHSL-serine 660 with different kinetics and different agonist efficacies within the same samples. There are notable similarities in the data obtained for pPeri-site 5 and pHSL-serine 660, including more rapid phosphorylation of these sites, and equal efficacies of FSK and L-γ-MSH at the 5 and 20 minute time points, and similarities between the data obtained for pPeri-site 6 and pHSL-serine 563 (phosphorylation of these sites occurred more slowly, and L-γ-MSH was a stronger agonist than FSK). Results with similar kinetics and relative magnitudes of responses to FSK and L-γ-MSH were also obtained in two additional time course experiments.

### Differential Phosphorylation of Perilipin and HSL in Response to a Panel of Lipolytic Agents

In related experiments, 3T3L1 cells were exposed to a panel of lipolytic agents, which consisted of 0.01 µM isoproterenol, 0.1 µM isoproterenol, 1 µM FSK, 6 µM FSK, and 0.2 µM L-γ-MSH, for 20 minutes, then fixed and labeled in a manner identical to the experiments described above.

**Figure 8:**
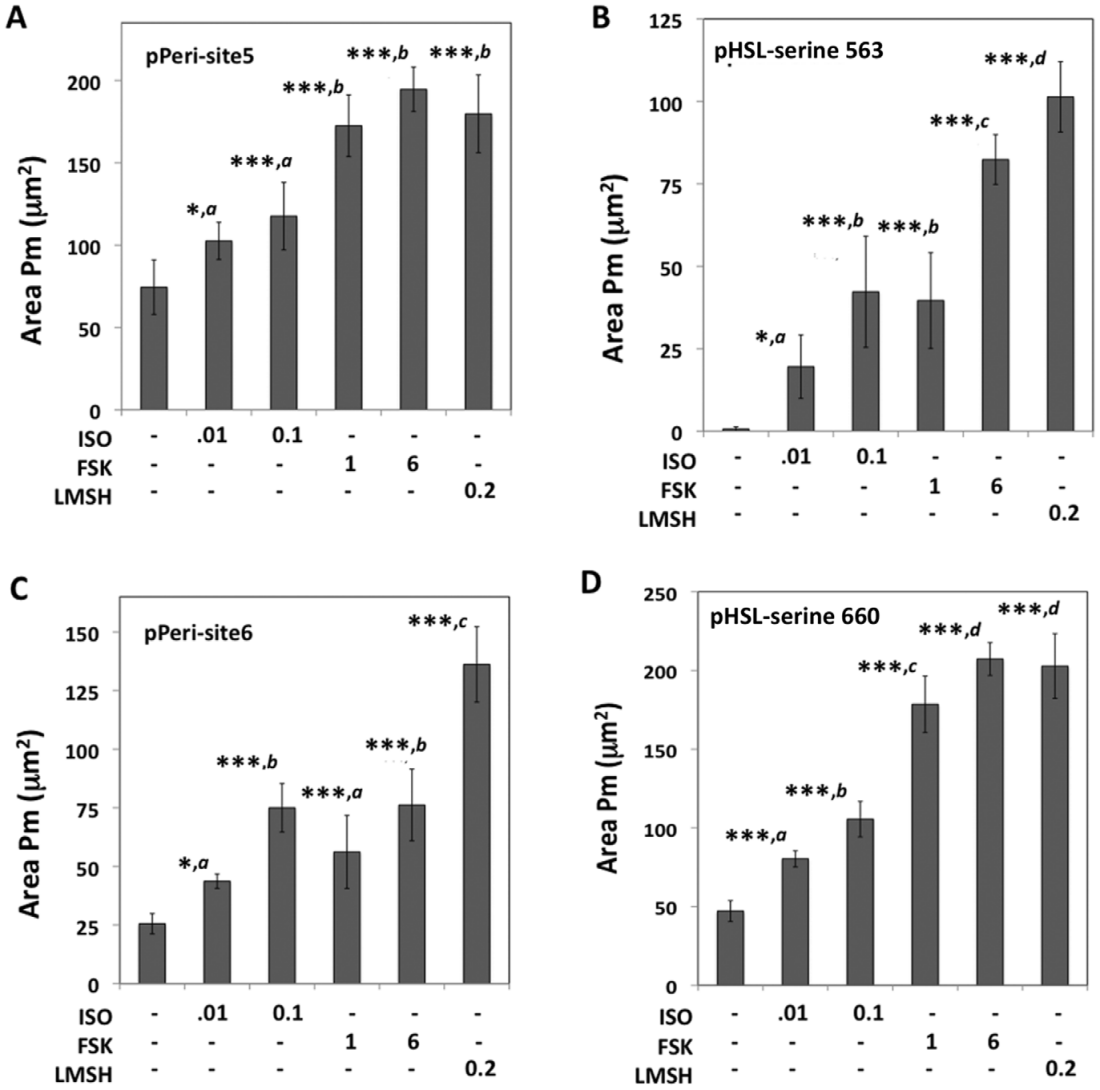
Differential phosphorylation of perilipin 1A and HSL in response to a panel of lipolytic agents. 3T3L1 adipocytes were exposed to the indicated concentrations (µM) of isoproterenol (ISO), FSK, and L-γ-MSH for 20 minutes, then fixed and labeled either PKA-site 5+ pHSL-serine 563 or PKA-site 6+ pHSL-serine 660. The cells were imaged with a 20X objective, 4 images/well, yielding an average of 360 cells/well. Area Pm values are shown for (A) PKA-site 5 (B), pHSL-serine 563, (C) PKA-site 6, and (D), pHSL-serine 660, respectively. Each bar represents the mean ± SD for n = 8 wells. * p<0.05 vs. control (ANOVA followed by Tukey’s test procedure); *** P<0.001 vs. controls. Within each panel, conditions distinct from one another at the p<0.05 level are also designated (*a,b,c,d*).

For pPeri-site 5 (Figure 8A), Area Pm for control cells averaged approximately 70 µm^2^. The low and high concentrations of isoproterenol increased Area Pm by approximately 30%, or 50%, respectively. The results for both concentrations of FSK, and for L-γ-MSH were similar, with each agent increasing Area Pm by approximately 2.5-fold.

For pHSL-serine 563 (Figure 8B), there was negligible labeling of the control cells. The low and high concentrations of isoproterenol increased Area Pm to approximately 20 µm^2^ and 42 µm^2^; both of these values were significantly different from controls and from each other. For FSK, the lower concentration increased Area Pm to about 40 µm^2^ whereas the high dose increased Area Pm to about 80 µm^2^. L-γ-MSH increased Area Pm to approximately 103 µm^2^, which was the greatest observed effect of the treatments, and the effect was significantly increased relative to the data for the high dose of FSK.

For pPeri-site 6 (Figure 8C), Area Pm averaged 25 µm^2^ for control cells. Exposure to the low and high concentrations of isoproterenol increased Area Pm by 2-fold and 3-fold, respectively. The low and high concentrations of FSK elicited approximately 2.5-fold and 3-fold increases in Area Pm. L-γ-MSH increased Area Pm approx. 5-fold, which was the strongest response observed in these experiments.

For pHSL-serine 660 (Figure 8D), Area Pm averaged 48 µm^2^ for controls. Exposure to the low and high doses of isoproterenol increased Area Pm by 70% and 2-fold, respectively. For FSK, the low and high concentrations elicited 3.8-fold and 4.3-fold increases in Area Pm, whereas L-γ-MSH increased Area PM by 4.2-fold.

In the above experiment, the response patterns obtained for pPeri-site 5 and pHSL-serine 660 are strikingly similar with perfect agreement in the rank order effectiveness of the treatments. The response patterns for pPeri-site 6 and pHSL-serine 563 are also very similar to each other and are different from the response patterns for pPeri-site 5 and pHSL-serine 660.

### Experiments with Blocking Peptides

The close similarities between the results for pPeri-site 5 and for pHSL-serine660, and the similarity between the results for pPeri-site 6 and pHSL-serine 563 raised the possibility that the antibodies to phospho-perilipin may be labeling phospho-HSL, or vice versa. To test if these peptides would block labeling of the cells by the primary antibodies, cells were incubated with primary antibodies that were pre-incubated with blocking peptides corresponding to pPeri site 5, pPeri site 6, or pHSL serine 660.

**Figure 9:**
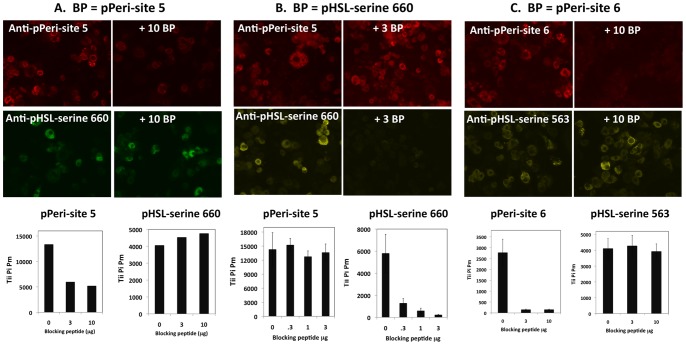
Tests for antibody specificity utilizing blocking peptides. 3T3L1 adipocytes were exposed to either 1 µM isoproterenol or 100 nM L-γ-MSH. Prior to labeling, primary antibodies were preincubated with the indicated amounts (µg) of blocking peptides corresponding to pPeri-site 5, pPeri-site 6, or pHSL-serine 660. Anti-phospho-perilipin 1A and anti-phospho-HSL antibodies were visualized in the red and far-red fluorescence channels, respectively. A, Results are shown for cells treated with isoproterenol for 15 minutes in which anti-pPeri-site 5 and anti-pHSLserine 660 were blocked with pPeri-site 5. Upper panels depict cell images. Lower bar graphs depict Tii Pi Pm data for pPeri-site 5 and pHSL-serine 660. B, Results are shown for cells treated with isoproterenol for 10 minutes in which anti-pPeri-site 5 and anti-pHSL-serine 660 were blocked with pHSL-serine 660. C, Results are shown cells treated with L-γ-MSH for 7 minutes in which anti-pPeri-site 6 and anti-pHSLserine563 were blocked with pPeri-site 6. For A, each bar represents a single well; for B and C, each bar represents the mean ± SD for n = 3 wells.

For 3T3L1 adipocytes treated with 1 µM isoproterenol, preincubation with the pPeri site 5 peptide reduced the overall image intensities resulting from labeling by the anti-pPeri site 5 antibody, and the Tii Pi Pm data parameter (which represents the protein labeling intensity, quantified on a per cell basis) (Figure 9A). Thus, binding of anti-pPeri site 5 antibody to its target is blocked by peptide corresponding to pPeri site 5, confirming the specificity of the antibody. In contrast, the pPeri site 5 peptide did not reduce image intensities or Tii Pi Pm resulting from labeling by the anti-pHSL serine 660 antibody (Figure 9A); thus, the anti-pHSL serine 660 antibody does not bind to pPeri site 5.

In a reciprocal experiment, also featuring 3T3L1 adipocytes treated with 1 µM isoproterenol, preincubation of anti-pPeri-site 5 antibody with the pHSLser660 peptide did not reduce overall image intensities or Tii Pi Pm obtained with the anti-pPeri-site 5 antibody (Figure 9B); in contrast, the pHSL serine 660 peptide strongly reduced the image intensities and Tii Pi Pm values obtained with the anti-pHSL-serine 660 antibody (Figure 9B). The results demonstrate that the anti-pPeri-site 5 antibody likely does not bind to pHSL serine 660 and confirms the specificity of the anti-pHSL-serine 660 antibody.

Finally, for 3T3L1 adipocytes treated with 100 nM L-γ-MSH, preincubation of anti-pPeri-site 6 antibody with a peptide corresponding to pPeri-site 6 strongly reduced image intensities and the Tii Pi Pm values obtained with anti-pPeri-site 6 antibody, but did not reduce overall image intensities or the Tii Pi Pm values obtained with the anti-pHSL-serine 563 antibody (Figure 9C). The results confirm the specificity of the anti-pPeri-site 6 antibody for pPeri-site 6, and demonstrate that the anti-pHSL-serine 563 antibody does not bind to pPeri-site 6.

Overall, the results with the blocking peptides confirmed the specificity of the phospho-perilipin and phospho-HSL antibodies for labeling their intended targets and indicate it is highly unlikely that the anti-perilipin 1A antibodies recognize HSL, or vice versa.

### Analysis of Perilipin 1A Phosphorylation via Western Blotting

**Figure 10:**
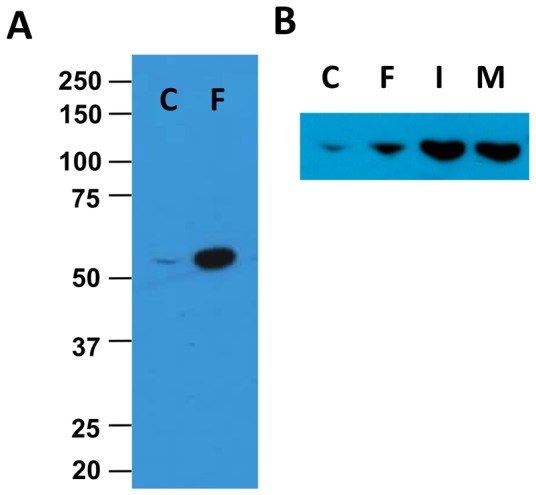
Phospho-perilipin 1A visualized via Western blotting. 3T3L1 adipocytes were exposed to lipolytic agonist for 5 minutes; whole cell lysates were then prepared and subjected to SDS-PAGE/Western blotting (20 µg protein/lane) utilizing the anti-pPeri-site 6 antibody. A, Results are shown for cells exposed to either control medium (C) or 6 µM FSK (F). B, Results are shown for a separate experiment in which cells were exposed to control medium, 6 µM FSK, 1 µM isoproterenol (I), or 100 nM L-γ-MSH (M).

To explore additional applications for the monoclonal antibodies against phospho-perilipin, we tested the ability of these antibodies to identify phospho-perilipin 1A by Western blotting. In our hands, anti-pPeri-site 5 did not visualize specific bands on Western blots. In contrast, anti-pPeri-site 6 labeled a single band, which migrated at approx. 60 KDa, which is consistent with the molecular weight of perilipin 1A, and the intensity of this band was strongly increased in samples for 3T3L1 cells treated with forskolin (Figure 10A). Furthermore, for experiments in which adipocytes were treated for 5 minutes with control, 6 µM FSK, 1 µM isoproterenol, and 100 nM L-γ-MSH, the bands from cells treated with isoproterenol and L-γ-MSH recognized by the anti-pPeri-site 6 antibody were more intense than the band for cells treated with FSK (Figure 10B). The observation is consistent with the previous observations that L-γ-MSH is a stronger agonist than FSK for phosphorylation of perilipin at PKA-site 6, as quantified via the microscopy methods. The strong response to isoproterenol, vs. FSK is somewhat unexpected considering the results from the microscopy assay. However, in the experiment for the Western blot, the cells were treated with a higher concentration of isoproterenol than utilized in the microscopy experiment. Results similar to those shown in Figure 10 were obtained in 2 additional experiments with anti-pPeri-site 6. In all, the results confirm the anti-pPeri-site 6 antibody recognizes phosphorylated perilipin 1A and this antibody is compatible with Western blotting.

## Discussion

The goal of this study was to examine the phosphorylation of perilipin 1A for comparison to HSL phosphorylation, at the initiation of lipolysis. Towards this purpose, we developed novel monoclonal antibodies (anti-pPeri-site 5 and anti-pPeri-site 6), which specifically recognize perilipin 1A phosphorylated at either PKA site 5 or PKA site 6, respectively. The specificity of these antibodies was confirmed in several ways. First, these antibodies recognized the synthetic phospho-peptides corresponding to the perilipin 1A PKA phosphorylation sites used in immunizing the mice, by ELISA. In subsequent experiments, these antibodies labeled ectoptically expressed perilipin 1A in FSK/IBMX-treated HeLa cells with no labeling of other proteins endogenous to these cells. Also, the labeling of ectopic perilipin 1A in HeLa cells was eliminated by single point mutations in perilipin where alanine was substituted for serine at the target site. The labeling by each antibody was also increased by FSK, an agent known to stimulate PKA-mediated phosphorylation of perilipin. The anti-pPeri-site 5 and anti-pPeri-site 6 antibodies labeled the edges of lipid droplets, consistent with the cellular localization pattern for perilipin 1A, and the labeling patterns were coincident with labeling by GP29, a well-established guinea pig polyclonal antibody to perilipin 1A. Finally, for FSK-treated human adipocytes, an siRNA specific for perilipin 1A down-regulated labeling by anti-pPeri-site 5, anti-pPeri-site 6, and GP29 in an identical manner. As pPeri-site 5 and pPeri-site 6 are associated with the carboxyl terminal of the protein, the antibodies are specific perilipin 1A, which is the full-length form of perilipin [3]. Notably, anti-pPeri-site 6 has recently been utilized by others to characterize the control of lipolysis in a variety of contexts; findings from these studies include increased lipolysis and phosphorylation of perilipin 1A associated with adaptive thermogenesis [30], increased lipolysis and phosphorylation of perilipin 1A on downregulation of the Berardinelli-Seip Congenital Lipodrystrophy 2/Seipin (BSCL2) protein [31], diminished β-adrenergic-induced lipolysis and phosphorylation of perilipin 1A on downregulation of CD36 [32], and increased lipolysis with unchanged phosphorylation of perilipin 1A in response to Fas [33].

Our results demonstrate that both perilipin 1A and HSL have sites that are phosphorylated at different rates in response to lipolytic agents and these sites are phosphorylated in response to lipolytic agents, in a strikingly similar manner. For example, perilipin 1A PKA-site 5 and HSL-serine 660 were phosphorylated rapidly and with equal efficacy in 3T3L1 adipocytes exposed to either FSK or L-γ-MSH. In contrast, perilipin 1A PKA-site 6 and HSL serine 563 were phosphorylated more slowly, and L-γ-MSH was a markedly stronger agonist for this effect than FSK. Indeed, when cells were exposed to an extended panel of lipolytic agents, the pattern of results for pPeri-site 5 was virtually identical to that for pHSL-serine 660, whereas the pattern of results for pPeri-site 6 were very similar to that for pHSL-serine 563. These similarities were not due to recognition of HSL by the antibodies raised to perilipin 1A or recognition of perilipin 1A by the antibodies raised to HSL, as the antibodies were selectively blocked by peptides that correspond to their intended targets.

The differential phosphorylation of HSL (more rapid at serine 660 vs. serine 563 in response to FSK) was first observed by Martin and colleagues [16], who hypothesized this might be accomplished by separate pools of PKA anchored to different cellular locations (e. g., the cytoplasmic side of the plasma membrane, or the edges of the lipid droplets). While our data is in agreement with the likely role of PKA-anchoring proteins such as Optic Atrophy-1 [34] in orchestrating the phosphorylation of perilipin 1A and HSL, an important distinction is perilipin 1A remains associated with lipid droplets during the initiation of lipolysis, whereas HSL undergoes a translocation step. Thus, perilipin 1A is differentially phosphorylated in a manner similar to HSL, even though perilipin 1A remains stationary. Since perilipin 1A is stationary, the differential phosphorylation of perilipin 1A is unlikely due to pools of PKA sequestered in different cellular locations by PKA-anchoring proteins, as suggested for HSL [16].

Consideration of the amino acid sequences of the PKA phosphorylation sites on perilipin 1A and HSL leads us to suggest an alternative hypothesis to account for the differential phosphorylation of these proteins. The consensus sequence for PKA is considered to be R(R/K)X(S/T) [35]. While the sequences surrounding perilipin 1A PKA site 5, perilipin 1A PKA site 6, and HSL serine 660 conform to this sequence, the sequence surrounding HSL-serine 563 does not, as there is no arginine at the P-3 position (Figure 1B). Even for phosphorylation sites that conform to the consensus sequence, it is well known that different sequences are phosphorylated more readily by PKA than others. For example, peptides with arginines at both the P-2 and P-3 positions (such as perilipin PKA site 5 and HSL serine 660) have a higher affinity for PKA than peptides containing only a single arginine at either position, (such as perilipin PKA site 5 and HSL serine 563) [36], [37], [38]. Surveys of the human genome suggest 80% of sequences represented by RRXS are physiological substrates for PKA. In contrast, only 48% of sequences represented by RKXS are PKA substrates, likely reflecting the lower affinity of the RKXS sequences for PKA [39], [40]. The sequences shown for perilipin 1A PKA sites 5 and 6, and for HSL serine 563 and serine 660 are conserved across a variety of mammals, including human, mouse, rat, and pig. We hypothesize perilipin PKA 1 site 5 and HSL-serine 660 have a higher affinity for PKA, and are thus phosphorylated more readily than perilipin 1A PKA site 6 and HSL-serine 563, due to the presence of arginines in both the P-2 and P-3 positions of perilipin 1A PKA site 5 and HSL-serine 660. The fact that such sequences are evolutionarily conserved suggests both high- and low-affinity sites for PKA phosphorylation are likely required for the functions of perilipin 1A and HSL.

Indeed, perilipin 1A PKA-site 5 and site 6 have previously been linked to different physiological processes (lipid droplet-dispersion for PKA site 5 [12], and ATGL-dependent lipolysis for PKA site 6 [13]). It is intriguing that lipid droplet dispersal, which requires several hours to manifest, is regulated by phosphorylation of perilipin 1A PKA-site 5, a site that is rapidly phosphorylated. Furthermore, the matching results for phosphorylation of perilipin 1A PKA-site 5 and HSL-serine 660 by the panel of lipolytic agents suggest that these phosphorylation events may be related, but HSL has not been implicated in lipid droplet dispersal. The results emphasize that much remains to be elucidated about the relationships between phosphorylation of perilipin 1A and HSL and the orchestration of the lipolytic response.

The reason for the greater effectiveness of L-γ-MSH compared to FSK at inducing the appearance of perilipin 1A PKA-site 6 and pHSL-serine 563 is unclear. One possibility is these sites are substrates for an additional kinase, other than PKA, which is activated by L-γ-MSH. Cyclic-GMP-dependent Protein Kinase (PKG), might be a candidate for this as it shares substrate specificity with PKA and is present in 3T3L1 adipocytes [41]. Furthermore, atrial natriuretic peptide likely activates lipolysis in human adipocytes by PKG-mediated phosphorylation of HSL at serine 660 [25]. However, L-γ-MSH is not known to activate PKG.

High content analysis methods provide several advantages over traditional immunofluorescence labeling and analysis techniques. The high throughput plating configuration enables processing of a larger number of samples per experiment than is practical with procedures in which cells are plated on coverslips, which must be handled individually and mounted on slides. Secondly, the image analysis algorithms objectively analyze each cell imaged, providing quantitative data for hundreds of cells per condition. High content analysis permits discrimination between effects subtly different between experimental treatments and greater resolving power for statistical analysis. While high content analysis strategies have most commonly been utilized in high throughput screening applications, our study demonstrates the value of utilizing HCA to examine the early events associated with activation of lipolysis, a pathway of key importance to obesity and the metabolic syndrome. Our data suggests future directions for research such as testing the hypothesis that differential phosphorylation of perilipin 1A and HSL depends upon the amino acid sequences flanking the phosphorylation sites, and to pursue further elucidation of the mechanisms by which L-γ-MSH elicits phosphorylation of perilipin 1A and HSL.
